# Association between Dental Erosion and Diet in Brazilian Adolescents Aged from 15 to 19: A Population-Based Study

**DOI:** 10.1155/2014/818167

**Published:** 2014-02-13

**Authors:** Yêska Paola Costa Aguiar, Fábio Gomes dos Santos, Eline Freitas de Farias Moura, Fernanda Clotilde Mariz da Costa, Sheyla Marcia Auad, Saul Martins de Paiva, Alessandro Leite Cavalcanti

**Affiliations:** ^1^Department of Dentistry, Faculty of Dentistry, State University of Paraiba, Avenida das Baraunas, S/N, Bodocongo, 58429-500 Campina Grande, PB, Brazil; ^2^Department of Pediatric Dentistry and Orthodontics, Faculty of Dentistry, Universidade Federal de Minas Gerais, Avenida Antônio Carlos 6627, Campus Pampulha, 31270-901 Belo Horizonte, MG, Brazil

## Abstract

Dental erosion is a pathological condition resulting from the irreversible dissolution of the mineralized portion of the teeth, being recognized in modern society as an important cause of loss of tooth structure. The aim of this study was to assess the prevalence and its association with diet in Brazilian adolescents of Campina Grande, PB, Brazil. A population-based study was conducted on a stratified sample of 675 adolescents aged from 15 to 19 of both sexes using the index proposed by O'Sullivan. Dental examinations were performed by two calibrated dentists (kappa = 0.82). The significance level adopted was 5%. The prevalence of dental erosion was 21%, and the upper central incisors and lateral incisors were the most affected elements, with 50.5% and 40.2%, respectively. The buccal surface showed greater impairment (51.4%) and 67.8% of teeth with dental erosion had more than half of the surface of affected area. Most damage was on the enamel (93.5%). There were no statistically significant differences between the occurrence of dental erosion and gender, age, socioeconomic status, self-reported ethnicity, and diet. There was high prevalence of dental erosion in its early stages among adolescents and there were no significant differences in the frequency of the consumption of foods and beverages and the presence of dental erosion.

## 1. Introduction

Dental erosion is an irreversible pathological condition manifested by the substantial loss of hard dental tissue due to chemical dissolution caused by acids without bacterial involvement [1]. The chemical events that lead to softening and eventual loss of the tooth surface are complex and well understood [2], having as a dominant factor dental exposure to low pH [3, 4] of substances that may be of intrinsic or extrinsic origin [5].

The etiology of dental erosion is varied, which can be idiopathic or caused by a known acid source [6], from the interaction of complex causes [7]. In this sense, it is observed that this disease has multifactorial background [8] in which individual factors and lifestyle have great relevance on its development [9, 10].

Some authors have observed high consumption of potentially erosive beverages such as soft drinks and fruit juices among young people [11, 12]. Thus, the involvement of children, adolescents, and adults has been attributed to changes in eating habits, with increased intake of processed products [13], which identifies diet as an important source of acids that contributes to the development of this disease [12–14].

During the last decade, there has been a significant increase in the prevalence and severity of erosive tooth wear, particularly in children and adolescents [15, 16]. Thus, data on the prevalence of dental erosion have attracted increasing attention from the dental community [11, 17]. However, a factor that makes it difficult to estimate the prevalence and comparison between epidemiological studies is the variation of indexes used and severity limits adopted by each index [5, 18–21], as well as different ages, groups of teeth, and faces assessed by researchers [15].

Different studies performed with adolescents in Greece [22], Turkey [23], and Australia [24] have reported prevalence of dental erosion of 51.6%, 52.6%, and 68%, respectively. In Brazil, several studies have been carried out [9, 14, 15, 25–28] with prevalence ranging from 1.8% [27] to 34.1% [15]. Erosive lesions preferentially occur on the palatal surfaces of upper anterior teeth and on the occlusal surfaces of the first lower molar teeth [10].

Considering that Brazil has great socioeconomic and cultural diversity [14], being a country of continental dimensions, data on the subject are still scarce in the literature [17], especially with regard to studies with adolescents in the northeastern region of the country. Therefore, this study aimed to determine the prevalence of dental erosion and its association with diet among adolescents aged 15–19 years.

## 2. Materials and Methods

### 2.1. Study Population

A population-based study was carried out in Campina Grande, Brazil. The city is the capital of the state of Paraiba and it has approximately 385,213 inhabitants. All twenty state urban schools with adolescents within the age group mentioned above enrolled in the day shift participated in the survey. Subjects were included if they were present on the day of data collection with the ICF (informed consent form) signed by parents.

There were 6514 students in the age group being studied enrolled in state schools in 2012. Sampling was of probabilistic type by cluster in a stratum (classes). It was used for the sample size calculation at confidence interval level of 95% and a 50% of disease prevalence. A correction factor of 1.7 was applied to compensate for the design effect. The minimal sample size, needed to satisfy the requirements, was estimated at 618 individuals. An additional 20% were added in the study to compensate for potential refusals, giving a total sample of 743 adolescents. To ensure sample representativeness, distribution was performed proportionally to the number of students per school [28].

### 2.2. Training and Calibration Process

Adolescents were examined by two dentists previously trained and calibrated. The calibration process consisted of theoretical activities with discussion of criteria adopted for the diagnosis for the different levels of dental erosion based on the diagnosis of clinical intraoral images [15, 26, 28]. In addition, 20 individuals were examined twice by the same researchers [17], with an interval of 10 days between each examination. A gold standard examiner (S.M.A.) managed the calibration process. Agreement analysis used Cohen's kappa coefficient on a tooth-by-tooth basis. The individuals included in the calibration process did not take part in the main study. Data from this pilot study demonstrated that there was no need to modify the methods previously proposed.

### 2.3. Dental Examinations

Dental examinations were conducted in a school room with the adolescent seated in front of the examiner. Data were recorded by a trained assistant. The dental exam occurred under artificial illumination (Petzl Zoom head lamp, Petzl America, Clearfield, UT) [14, 26, 29] and with dental mirrors and periodontal probes (Trinity, Campo Mourao, PR, Brazil). Sterile gauze pads were used to clean and dry the teeth.

Dental erosion was evaluated according to location, severity, and area affected by the condition [6] adapted for use in the four upper incisors [25] and the first molars [17, 26]. Adolescents with braces or who had enamel hypoplasia [17] were excluded from the study.

### 2.4. Nonclinical Data Collection

Data were collected through questionnaire followed by clinical oral examinations. The questionnaire was previously validated [14] and applied to obtain information about the intake of food associated with dental erosion and data on family income and self-reported ethnicity.

The consumption of foods and beverages was dichotomized into high (once a day and more than once a day) [8] and low (never, once a week, and two to four times a week) [9, 14, 30]. Family income was classified as less than two minimum wages and greater than or equal to two minimum wages and race as white or nonwhite [28].

### 2.5. Data Analysis

Data analysis involved descriptive statistics (frequency distribution) and analytic statistics. To test the association between the occurrence of dental erosion and diet, a process of bivariate analysis was conducted, using the exact versions of the nonparametric Pearson's chi-squared test or Fisher's exact test. The level of statistical significance was set at 5% with a confidence interval of 95%.

### 2.6. Ethical Aspects

This study followed ethical guidelines recommended by the Brazilian legislation and was approved by the Human Research Ethics Committee of the State University of Paraiba. All participants/guardians signed the informed consent form.

## 3. Results

Kappa values calculated to assess intra- and interexaminer variability were 0.74 and 0.82, respectively. Of the 743 subjects estimated to compose the sample, 675 were examined (273 males and 402 females), representing a response rate of 90.8%.

The prevalence of dental erosion was 21%, with no significant differences between gender, age, income, and self-reported ethnicity of participants (*P* > 0.05). More than half (55.4%) of adolescents belonged to the families whose incomes were lower than two minimum wages and most adolescents reported themselves as nonwhite (78.5%), as shown in Table 1.

From a total of 4681 teeth (14,043 faces) examined, 463 (9.9%) showed dental erosion. A greater number of erosive lesions were observed in the upper central incisors, followed by upper lateral incisors and upper and lower first molars (Table 2).

In relation to dental erosion severity, most affected faces (60.25%) exhibited enamel with “satiny” appearance without loss of surface contour, which indicates initial stages of loss of tooth structure. Losses of enamel beyond the dentinoenamel junction and pulp exposure were not observed in any of the subjects. Concerning the location of lesions, the buccal surface was the most affected (51.4%) followed by multiple surfaces (21.4%). More than half the surface of the affected areas was observed in 67.8% of teeth affected by dental erosion (Table 3).

Table 4 shows the frequency of consumption of various foods and the occurrence of dental erosion. Among adolescents with high consumption, there was a higher intake of pure coffee or with milk. There were no significant differences in the frequency of the consumption of foods and beverages and the presence of dental erosion.

## 4. Discussion

The high intra- and interexaminer response rate observed indicates that the external validity of this study was good. The main reasons for nonparticipation of adolescents were no-show on the day of exam [14, 15] and lack of consent form signed by parents [25, 28].

The use of index teeth in the epidemiological investigation of dental erosion, as used in this work, has been performed frequently [1, 14, 15, 25, 26, 31–33]. This tool has been considered appropriate since these teeth are exposed in the mouth for a longer period of time compared to other teeth, being more susceptible to the action of etiological factors [25, 34]. Therefore, the use of index teeth does not substantially influence the prevalence of dental erosion [17], reflecting the situation of the study population. Moreover, the clinical examination should be carried out systematically by applying a simple and accurate index [35].

In the present work, the O'Sullivan index was adopted [6], which was adapted for use on upper incisors [25] and the first permanent molars [17, 26] since the presence of cuppings in the cusps of molars in adolescents is one of the most common presentations of dental erosion [36]. This index has been used in epidemiological studies in Germany [37], China [38], and Brazil [17, 25, 26, 28, 34] and is considered simple, applicable to young, deciduous, and permanent dentition and can be used for the diagnosis of dental erosion of any etiology [6].

The methodological differences have made the direct comparison between studies difficult [5, 8, 15, 18–21]. Regarding the prevalence of dental erosion, there is wide variability among different populations and location [20].

The occurrence of this condition in Brazil has shown great variability. In the present study, the prevalence of dental erosion was 21%, similar to that described by other researchers [9, 34]. However, compared with other studies conducted in the southeastern region of the country, this prevalence was lower than 26% observed in adolescents aged 12 [26] and 34.1% found in adolescents aged from 13 to 14 years [14, 15]. Similarly, there is great variability in the prevalence of dental erosion in different countries: 5.5% in the United States [39] and 68% in Malaysia [40]. In Europe, its occurrence ranges from 14% among Danish adolescents [41] to 59.7% among English adolescents [31].

In this study, boys showed higher experience of dental erosion compared to girls, but this difference was not statistically significant, as observed in previous studies [1, 15, 25, 26, 28, 33]. However, there are reports [8, 29, 31–34] of statistically significant differences in relation to gender, with greater involvement of male subjects, which can be explained by greater degrees of consumption of cola drinks among male adolescents [29].

In this study, no differences related to age were found. This fact can be explained by the small age range (15–19 years) used, although adolescence is a time when dental erosion progresses rapidly [41]. Some authors also found no significant differences related to age [23, 38] and others observed prevalence twice as high when comparing this data in individuals aged between 12 and 15 years [29]. The literature shows that, for every one-year increase in age, the likelihood of an adolescent to have this disease increases by up to 1.3 times for all teeth and if the disease is not diagnosed early, it can develop in adulthood [32].

In terms of family income and prevalence of dental erosion, no statistically significant differences were found between subjects belonging to families with incomes below two minimum wages and those with incomes greater than or equal to this value, which has been previously observed [28]. Other studies have reported that socioeconomic status does not influence the occurrence of dental erosion [14, 15], although, in general, people from higher socioeconomic classes show higher prevalence of dental erosion [28].

However, it should be emphasized that this study was conducted with students from public schools, which do not require monthly tuition [14, 15], possibly covering a portion of the population with lower purchasing power, thus decreasing the socioeconomic contrast experienced by participants. However, some authors considered the type of school (public or private) as an indicator of social class, finding higher prevalence of erosion in private school students, with higher socioeconomic status [25]. Therefore, there is a lack of consensus in relation to the relationship between socioeconomic status and the occurrence of dental erosion.

As for ethnicity, no statistically significant difference was observed, confirming previous findings [28]. However, previous report revealed a higher proportion of white adolescents with dental erosion compared to Asians and explained this fact due to the exposure to different etiological and modifying factors [31].

A greater number of erosive lesions were observed in the upper incisors, which is in agreement with previous reports [15, 17, 28, 31, 38, 42]. Regarding the maxillary lateral incisors, the fact that the central incisors erupt before maxillary lateral incisors and therefore are exposed longer to the potential risk factors explains the higher occurrence of dental erosion in central incisors [15]. Only 3.3% of subjects exhibited erosion in the first permanent molars, which is close to 5% observed by others authors [15]. The low occurrence of dental erosion in these teeth can be explained by the difficulty of evaluating this tooth element in part of the sample (43.2%), since they showed extensive cavities or fillings, preventing the examination. On the other hand, the higher proportion of involvement of the first molars has been reported in other studies [8, 29, 43].

Regarding the severity of erosion and type of dental tissue affected, most lesions involved only the enamel, confirming previous findings [26, 29, 33, 34, 44]. Lesions confined to the enamel were observed in some studies [14, 15, 17, 24–26, 28]. The occurrence of dentin lesions was low, confirming previous findings [31, 44]. There was no pulp involvement in adolescents examined, similar to previous report [34].

If the aim is to stop the progression of dental erosion at the first phase, as observed in this study, early diagnosis is essential [5, 35, 43]. Oral health education is used to assist the population to understand dental erosion and its deleterious effects [45]. In cases of moderate erosion, the buccal and lingual surfaces of the maxillary anterior teeth show up smooth and shiny, with the loss of some anatomical features [7]. The appearance of silky and smooth surface with no perikymata and intact enamel along the gingival margin are some typical signs of enamel erosion on these surfaces [35].

Similar to other studies, the buccal surface of the upper incisors was the most affected [17, 22, 26, 28, 46]. This condition may result from frequent exposure to acidic beverages [26, 47]. However, some studies show the palatal surface as the most affected [15, 19, 25, 29, 31, 42, 43, 47]. The involvement of the palatal surface has been described as a consequence of the exposure of this surface to low pH of vomiting and gastroesophageal reflux [47] and to the tongue activity in this region soon after ingestion of soft drinks [29]. The controversy on this issue may be explained by the different forms of erosion assessment, added to the fact that these studies were performed in patients with different eating habits, which can affect the tooth surfaces in different ways and with different severity patterns [34].

More than half of surfaces of affected areas were observed in most of the teeth affected by dental erosion, corroborating previous findings [25, 26, 38]. However, some researchers found less than half of surfaces of sides affected by erosion [17, 28]. The involvement of more than half of surfaces affected by this disease suggests that these adolescents may have been exposed to high levels of risk factors for erosion or for a relatively long time [25].

The influence of diet on the development of dental erosion has been reported in the literature [4, 8, 11–14, 23, 30, 45]. Beer and milk are products essentially nonerosive; however, products with high concentration of acid (vinegar, lemon juice, and products containing lactic acid) are very erosive. Others are considered products of intermediate erosive potential (fruit juice, soft drinks, sports drinks, and tea) [4].

In this study, no associations between dietary habits and dental erosion were found, which is in agreement with other studies [1, 9, 28, 37]. However, some authors found positive association between diet and erosion [8, 14, 30, 38]. Thus, individual modifying factors such as the protective contribution of saliva through its flow rate and its buffering ability [48] as well as the extrinsic and intrinsic multifactorial characteristics of acids involved in the dental erosion etiology perhaps may explain the lack of association between diet and the occurrence of dental erosion observed in this work.

Due to the cross-sectional characteristic of this study, no conclusions could be drawn about the development of dental erosion in the study subjects. In addition, the use of index teeth to minimize the time of clinical examination is a condition that does not allow comparing the severity of dental erosion among all the elements present in the mouth of patients. Moreover, the study reflects the situation of adolescents enrolled in public schools in the city of Campina Grande, Brazil, so that data on higher income population possibly enrolled in the private school system were not obtained. Another limitation concerns the fact that some data were obtained from reports from adolescents and therefore subject to recall biases [22].

Since tooth erosion is a condition with high prevalence, early diagnosis will facilitate the approach targeted to the patient due to the multifactorial nature of the tooth exposure to acids, suggesting the monitoring of lesions found and the implementation of educational and preventive measures before major damage is observed.

## 5. Conclusion

Dental erosion is present in the adolescent population, regardless of gender, age, socioeconomic status, and ethnicity in its early stages. The upper central incisors and upper lateral incisors are the most affected elements and most lesions were located on the enamel. There was no association between dietary habits and occurrence of dental erosion.

## Figures and Tables

**Table 1:** Occurrence of dental erosion according to gender, age, income, and self-reported ethnicity.

Dental erosion							*P* value*
	Presence		Absence		Total	
	*n*	%	*n*	%	*n*	%
Gender							
Male	61	22.3	212	77.7	273	100	0.492
Female	81	20.1	321	79.9	402	100	
Age							
15	37	21.9	132	78.1	169	100	0.654
16	50	22.4	173	77.6	223	100	
17	39	21.7	141	78.3	180	100	
18	12	14.6	70	85.4	82	100	
19	4	19.0	17	81	21	100	
Family income							
<2 MW	78	20.9	296	79.1	374	100	0.897
≥2 MW	64	21.3	237	78.7	301	100	
Ethnicity							
White	31	21.4	114	78.6	145	100	0.909
Nonwhite	111	20.9	419	79	530	100	
Total	**142**	**21.0**	**533**	**79.0**	**675**	**100**	

*Chi-square test, MW (minimum wage of R$ 622.00 in the year 2012.

**Table 2:** Number of teeth examined and number (%) of teeth affected by dental erosion.

Tooth	Number of teeth examined*	Number of teeth with erosion	% with erosion
11	642	117	18.2
12	649	98	15.1
21	643	117	18.2
22	651	88	13.5
16	574	13	2.3
26	602	9	1.5
36	463	13	2.8
46	457	8	1.8
Total	**4681**	**463 **	**10.0**

*Teeth with restorations and extensive caries were not evaluated.

**Table 3:** Characteristics of dental erosion in relation to the number of teeth affected.

Tooth	11		12		21		22		16		26		36		46	
	*n*	%	*n*	%	*n*	%	*n*	%	*n*	%	*n*	%	*n*	%	*n*	%
*Severity *																
Enamel with “satiny” appearance (without loss of surface contour)	77	65.8	64	65.3	74	63.2	50	56.8	5	38.5	4	44.4	3	23.0	2	25.0
Loss of enamel (with loss of surface contour)	36	30.8	27	27.5	39	33.3	34	38.6	5	38.5	2	22.2	6	46.1	5	62.5
Loss of enamel with dentin exposure (visible JAD)	4	3.4	7	7.1	4	3.4	4	4.5	3	23.1	3	33.3	4	31.0	1	12.5
*Location *																
Only buccal	70	59.8	55	56.1	67	57.3	43	48.9	0	0	1	11.1	1	7.7	1	12.5
Only lingual or palatal	11	9.4	14	14.3	12	10.2	13	14.8	0	0	0	0	0	0	0	0
Only occlusal or incisal	1	0.8	1	1.0	1	0.8	1	1.1	11	84.6	6	66.7	9	69.2	5	62.5
Buccal, occlusal, or incisal	5	4.3	2	2.0	5	4.3	4	4.5	0	0	0	0	0	0	0	0
Lingual and incisal or occlusal	6	5.1	5	5.1	8	6.8	5	5.7	1	7.7	0	0	0	0	0	0
Multiple surfaces	24	20.5	21	21.4	24	20.5	22	25.0	1	7.7	2	22.2	3	23.0	2	25.0
*Affected area *																
Less than half of the surface	44	37.6	32	32.6	44	37.6	24	27.3	2	15.4	2	22.2	1	7.7	1	12.5
More than half of the surface	73	62.4	67	68.4	73	62.4	64	72.7	11	84.6	7	77.7	12	92.3	7	87.5
Total	**117**		**98**		**117**		**88**		**13**		**9**		**13**		**8**	

**Table 4:** Sample distribution according to the frequency of consumption and presence of dental erosion in Brazilian adolescents.

Food or beverage	High consumption*		Low consumption**		*P* value***	Odds ratio	CI 95%	
	Erosion present	Erosion absent	Erosion present	Erosion absent			Minor	Major
	*n* (%)	*n* (%)	*n* (%)	*n* (%)				
Fruits	56 (22)	198 (78)	86 (20.5)	334 (79.5)	0.628	0.910	0.623	1.331
Regular soft drink	0	0	142 (21)	533 (79)	—	—	—	—
*Diet/light* soft drink	0	0	142 (21)	533 (79)	—	—	—	—
Yogurt	29 (19.9)	117 (80.1)	113 (21.6)	411 (78.4)	0.656	1.109	0.703	1.751
Artificial fruit juice	30 (17.9)	138 (82.1)	112 (22.2)	393 (77.8)	0.234	1.311	0.838	2.050
Isotonic beverage	1 (12.5)	7 (87.5)	141 (21.2)	523 (78.8)	0.547	1.887	0.230	15.466
Natural fruit juice	56 (22.4)	194 (77.6)	86 (20.4)	336 (79.6)	0.535	0.887	0.606	1.297
Ice tea	2 (28.6)	5 (71.4)	140 (21)	527 (79)	0.625	0.664	0.127	3.459
Energy drink	3 (15)	17 (85)	139 (21.3)	513 (78.7)	0.495	1.535	0.444	5.314
Milk	51 (21.6)	185 (78.4)	89 (20.4)	347 (79.6)	0.715	0.930	0.631	1.371
Flavored milk	28 (19.7)	114 (80.3)	113 (21.4)	416 (78.6)	0.670	1.106	0.696	1.757
Coffee	73 (20.9)	276 (79.1)	67 (20.9)	254 (79.1)	0.989	0.997	0.687	1.448
Coffee with milk	52 (20.2)	206 (79.8)	90 (21.6)	326 (78.4)	0.647	1.094	0.745	1.605
Pickles	1 (25)	3 (75)	141 (21)	530 (79)	0.845	0.798	0.082	7.731
Mustard	6 (33.3)	12 (66.7)	136 (20.7)	521 (79.3)	0.194	0.522	0.192	1.416
Ketchup	17 (22.7)	58 (77.3)	125 (20.8)	475 (79.2)	0.713	0.898	0.505	1.596
Vinegar	18 (26.9)	49 (73.1)	124 (20.4)	484 (79.6)	0.217	0.697	0.392	1.240

*Combination of frequencies “1** **x/day and ≥ 2** **x/day”; **combination of frequencies “never, 1** **x/week, and 2–4** **x/week”; excluding lost cases; ***chi-squared test.
